# Case Report: Perioperative acquired long QT syndrome secondary to severe hypokalemia from duodenal foreign body obstruction in a toddler

**DOI:** 10.3389/fmed.2026.1810553

**Published:** 2026-04-07

**Authors:** Jiaying Xin, Jianguo Wang, Jinsong Sun, Laizhu Zhang, Xuebo Bai

**Affiliations:** 1 Department of Anesthesiology, Affiliated Hospital of Jining Medical University, Jining, China; 2 Department of Pediatric Surgery, Affiliated Hospital of Jining Medical University, Jining, China

**Keywords:** acquired long QT syndrome, duodenal obstruction, hypokalemia, magnesium sulfate, perioperative management

## Abstract

This case report describes the perioperative anesthetic management of a 14-months-old male toddler who developed acquired long QT syndrome (aLQTS) secondary to severe hypokalemia resulting from duodenal foreign body obstruction. Intensive intravenous potassium supplementation and prophylactic magnesium sulfate administration were initiated to stabilize myocardial electrophysiology and reduce arrhythmic risk. The patient subsequently underwent successful surgical removal of the obstructing foreign body. Postoperatively, electrolyte levels normalized, and the QT interval returned to baseline. This case highlights the critical importance of vigilance for aLQTS and the risk of malignant arrhythmias in toddlers presenting with severe vomiting and hypokalemia. Early recognition and synergistic intervention are key to preventing torsades de pointes (TdP).

## Introduction

Acquired long QT syndrome (aLQTS) is characterized by delayed ventricular repolarization resulting from extrinsic factors that disrupt cardiac ion channel function, including medications, electrolyte disturbances, and structural heart disease. Electrolyte abnormalities, particularly hypokalemia, hypomagnesemia, and hypocalcemia, are well-recognized contributors to QT interval prolongation and predispose patients to malignant ventricular arrhythmias, most notably torsades de pointes (TdP) (1–3). In toddlers, severe vomiting and diarrhea can precipitate acute, profound hypokalemia. During the perioperative period, correction of these abnormalities must be undertaken with heightened vigilance, as rapid shifts in electrolyte balance can unmask or exacerbate aLQTS. However, in routine anesthetic practice, the risk of aLQTS in pediatric patients is often underestimated, representing a critical gap in perioperative awareness and management. This case report describes the perioperative management of a toddler with severe hypokalemia-induced aLQTS secondary to duodenal foreign body obstruction. The case highlights the importance of early recognition and demonstrates the effectiveness of prompt, synergistic potassium and magnesium supplementation in mitigating arrhythmic risk and preventing TdP.

## Case report

**FIGURE 1:**
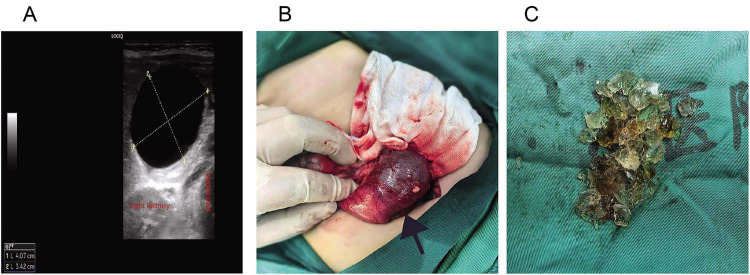
Diagnostic and operative findings of duodenal obstruction caused by a superabsorbent polymer ball. **(A)** Abdominal ultrasonography on admission reveals a cystic mass (4.1 cm × 3.7 cm × 3.4 cm, between calipers) in the proximal duodenum. **(B)** Intraoperative photograph after conversion to open laparotomy, demonstrating the dilated proximal duodenum (arrow) with an intraluminal foreign body (∼4.5 cm in diameter). **(C)** The retrieved foreign body, which was fragmented during extraction, is shown to be a fully expanded superabsorbent polymer ball.

**FIGURE 2:**
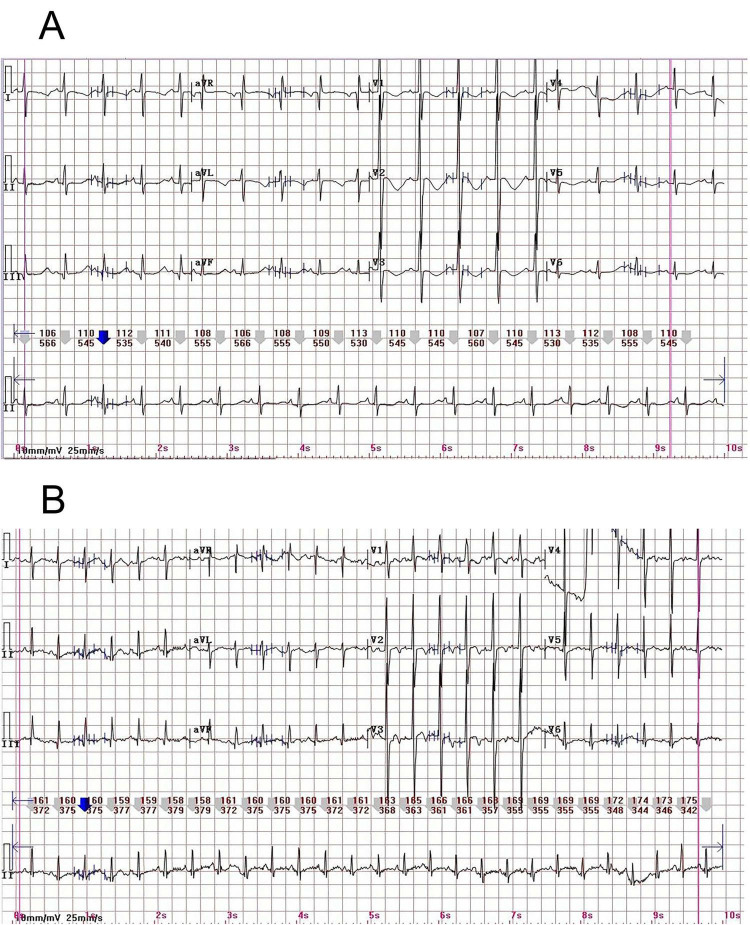
Pre- and postoperative ECG comparison. **(A)** Preoperative ECG. Manual measurement by an ECG specialist using the Fridericia formula yielded a prolonged QTc of 536 ms. **(B)** Postoperative ECG on day 5. The QTc interval had normalized to 422 ms, as confirmed by manual measurement using the same method.

A previously healthy 14-months-old toddler (height 90 cm, weight 10 kg) presented with a 7-days history of persistent vomiting unresponsive to symptomatic treatment at an outside hospital. There was no significant personal or family history of syncope, seizures, or sudden cardiac death. On admission, physical examination revealed lethargy, poor skin turgor, and a palpable firm mass in the upper abdomen. Vital signs showed heart rate 130 bpm, respiratory rate 26 breaths/min, and SpO2 93% on room air, likely due to dehydration-related hypoperfusion and lethargy, as chest auscultation was clear. Abdominal ultrasonography demonstrated a cystic mass measuring 4.1 cm × 3.7 cm × 3.4 cm in the proximal duodenum, consistent with intestinal obstruction (Figure 1A). Emergency laboratory investigations revealed profound hypokalemia (K^+^ 2.87 mmol/L; reference range 3.5–5.5), severe hypochloremia (Cl^–^ 68 mmol/L; 98–110), and hypomagnesemia (Mg^2+^ 0.73 mmol/L; 0.75–1.02). Additional abnormalities included hyponatremia (Na^+^ 132 mmol/L; 134–143), hypocalcemia (Ca^2+^ 2.0 mmol/L; 2.1–2.8), and metabolic alkalosis (HCO_3–_ 36.6 mmol/L; 22–29). An automated electrocardiogram (ECG) analysis indicated a corrected QT (QTc) interval of 499 ms. Subsequent manual measurement by an ECG specialist, using the Fridericia correction formula, confirmed marked QT prolongation with a QTc of 536 ms (Figure 2A). The patient was transferred emergently to the operating room for surgical intervention. Upon arrival, monitoring revealed a blood pressure of 94/60 mmHg, heart rate of 120 bpm, respiratory rate of 26 breaths per minute, and SpO_2_ of 92% on room air. General anesthesia was induced intravenously with propofol (20 mg), fentanyl (30 μg), and rocuronium (6 mg), followed by endotracheal intubation and mechanical ventilation in volume-controlled ventilation (VCV) mode. Initial ventilator settings included a tidal volume of 80 mL (approximately 8 mL/kg), respiratory rate of 22 breaths per minute, inspiration-to-expiration ratio of 1:1.5, and fraction of inspired oxygen (FiO_2_) of 0.5. End-tidal carbon dioxide (EtCO_2_) was continuously monitored and maintained between 35 and 45 mmHg. Anesthesia was maintained with inhaled sevoflurane, supplemented with intermittent boluses of fentanyl for analgesia. Subsequently, invasive arterial and central venous monitoring were established via a left radial arterial line and a right internal jugular central venous catheter, respectively. The first arterial blood gas (ABG) analysis, obtained at T_0_ (defined as the time of the first post-induction ABG), revealed life-threatening metabolic derangements: pH 7.71, PaCO_2_ 39 mmHg, K^+^ 1.7 mmol/L, Na^+^ 132 mmol/L, Cl 78 mmol/L, ionized calcium (iCa^2+^) 0.9 mmol/L, base excess of extracellular fluid (BEecf) + 29.7 mmol/L, and lactate 1.0 mmol/L. Given the life-threatening hypokalemia, the ABG analysis was immediately repeated on a fresh sample from the same arterial line. The repeat measurement confirmed the initial finding (1.7 mmol/L). Immediate interventions included: changing the maintenance intravenous fluid to 0.9% sodium chloride; initiating a central venous infusion of potassium chloride at 0.4 mmol/kg/h; and administering a slow intravenous bolus of magnesium sulfate (40 mg/kg; total 400 mg).

Intraoperative laparoscopic exploration revealed marked dilation of the proximal duodenum, highly suggestive of an intraluminal obstruction, prompting conversion to open laparotomy. During open exploration, a foreign body measuring approximately 4.5 cm in diameter was identified within the duodenal lumen (Figure 1B). A 1.5-cm longitudinal incision was made in the dilated duodenal segment to expose the obstructing object, which proved to be a fully expanded superabsorbent polymer ball, a toy material capable of dramatic expansion upon water exposure. The foreign body was fragmented and completely removed (Figure 1C). The total operative time was 100 min. Hemodynamic parameters remained stable throughout the procedure, and the perioperative corrected QT interval change (ΔQTc) fluctuated within ±25 ms. A postoperative arterial blood gas (ABG) analysis obtained at T_0_ + 2 h demonstrated a clear trend toward metabolic correction, although values had not yet fully normalized: pH 7.74, PaCO_2_ 31 mmHg, K^+^ 2.3 mmol/L, Cl 81 mmol/L, BEecf + 22.7 mmol/L, and lactate 2.6 mmol/L. Compared with the initial intraoperative values (pH 7.71, BEecf + 29.7 mmol/L, K^+^ 1.7 mmol/L), both metabolic alkalosis and hypokalemia showed significant improvement. The mild lactate elevation (2.6 vs. 1.0 mmol/L) likely reflected both preoperative hypovolemia and surgical stress, despite stable intraoperative hemodynamics.

**FIGURE 3:**
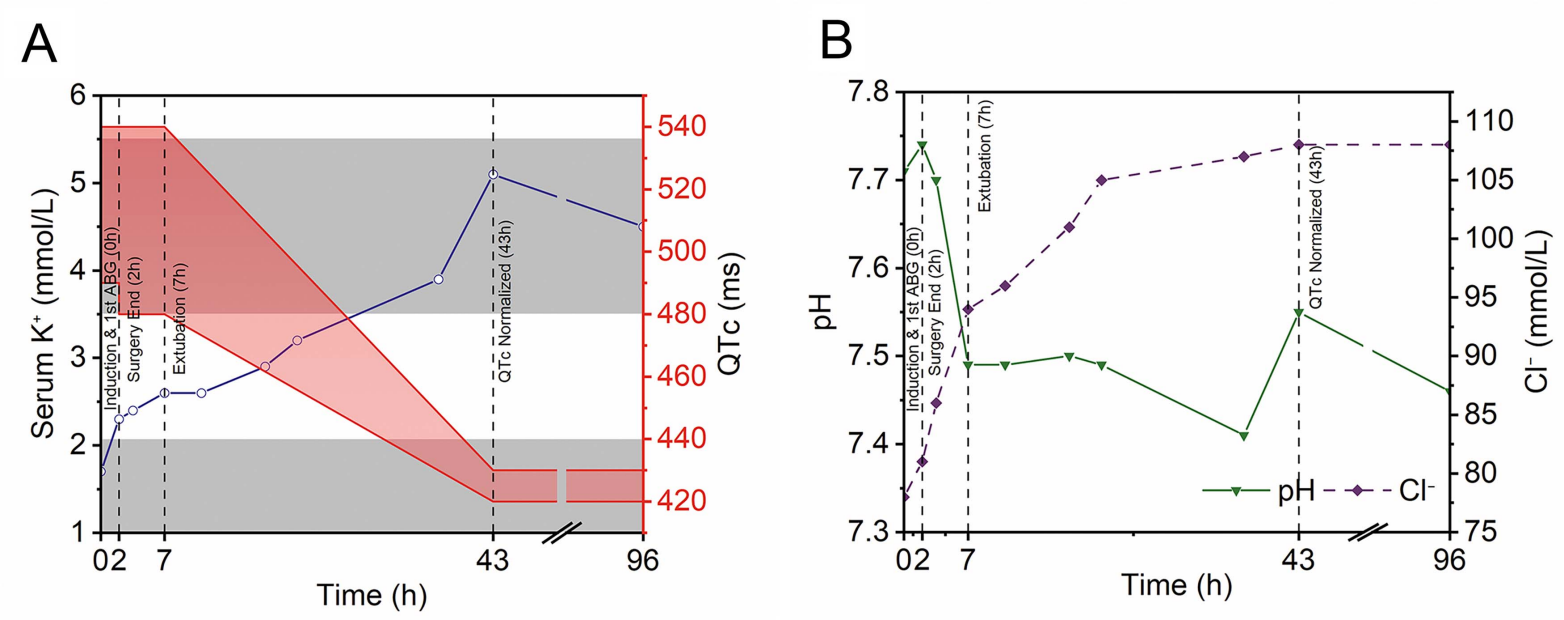
Temporal trends of key parameters during the perioperative period. Serial arterial blood gas analyses were performed at indicated time points (0, 2, 3.5, 7, 11.5, 18, 21.5, 37, 43, and 96 h). T_0_ (0 h) is defined as the time of the first post-induction ABG **(A)** Serum potassium (K^+^; blue solid line with circles) is plotted alongside the QTc interval (red shaded band). The QTc trend delineates four clinical phases: I. High-risk perioperative period (0–2 h; 490–540 ms), II. Early resuscitation (2–7 h; 480–540 ms), III. Stabilization (7–43 h; converging to 420–430 ms), and IV. Normalization (43–96 h; 420–430 ms). Key events, extubation (T_0_ + 7 h) and QTc normalization (T_0_ + 43 h), are marked. Gray shaded areas indicate normal ranges (K^+^: 3.5–5.5 mmol/L; QTc: <440 ms). **(B)** Arterial pH (green solid line with triangles) and serum chloride (Cl^–^; purple dashed line with diamonds). The gray bar indicates the physiological pH range (7.35–7.45).

Postoperatively, the patient was transferred to the Pediatric Intensive Care Unit (PICU). Continuous monitoring demonstrated a clear inverse relationship between rising serum potassium levels and progressive QTc shortening (Figure 3A). This electrophysiological stabilization paralleled gradual normalization of pH and chloride levels, indicating resolution of metabolic alkalosis (Figure 3B). The patient’s clinical recovery paralleled this improvement, with successful extubation at T_0_ + 7 h. By T_0_ + 43 h, the QTc interval had normalized, which is consistent with complete correction of the underlying metabolic disorder. On postoperative day (POD) 1, the serum magnesium level rose to 0.85 mmol/L. The patient was transferred back to the general ward on POD 4. A follow-up ECG on POD 5 revealed a QTc of 422 ms (Figure 2B), representing a significant decrease from the preoperative value. Electrolyte levels normalized by POD 6 (serum potassium 4.4 mmol/L; magnesium 0.88 mmol/L). The remainder of the recovery was uneventful, and the patient was discharged on POD 10.

## Discussion

This case report describes a pediatric patient who developed duodenal obstruction after ingestion of a superabsorbent polymer ball, leading to persistent vomiting, severe electrolyte disturbances, and secondary aLQTS. The favorable perioperative outcome can be attributed to early recognition of aLQTS, proactive implementation of antiarrhythmic risk-reduction strategies, and effective multidisciplinary coordination. The case is noteworthy because the patient presented with an imminent risk of malignant ventricular arrhythmia, underscoring the importance of anticipatory risk assessment and prompt intervention in anesthetic practice. The QT interval, measured from QRS onset to T wave end, reflects total ventricular depolarization and repolarization time. QT prolongation is strongly associated with TdP, ventricular fibrillation, and sudden cardiac death. Although perioperative QT prolongation is relatively uncommon, it is a recognized high-risk marker when present. Because the QT interval varies inversely with heart rate, QTc is used for clinical assessment. In pediatric patients from infancy through preadolescence, a QTc < 440 ms is generally considered normal, ≥460 ms indicates prolongation, and ≥500 ms is regarded as clinically high risk (4). Notably, automated ECG measurements of QT/QTc may be inaccurate in the presence of hypokalemia, tachycardia (heart rate > 100 bpm), or rhythm irregularity due to flattened T waves, T-U wave fusion, and limitations of heart rate correction algorithms. Therefore, to ensure accuracy in this case, an experienced cardiac electrophysiologist performed manual measurement using the Fridericia formula, which provides more reliable QTc correction during tachycardia. In accordance with 2023 CCS guidelines (3), all manual QTc measurements were obtained from Lead II using the Fridericia formula. The end of the T wave was defined by the tangent method, excluding U waves. Each reported value represents the mean of 3–5 consecutive beats, with key measurements confirmed by a consulting pediatric cardiologist.

In this patient, the preoperative QTc was markedly prolonged to 536 ms in the setting of hypokalemia (K^+^ 2.87 mmol/L) and hypomagnesemia (Mg^2+^ 0.73 mmol/L). After anesthetic induction (T_0_), arterial blood gas analysis revealed critical hypokalemia (K^+^ 1.7 mmol/L), and QTc values remained within a high-risk range. Following definitive surgical management and systematic correction of electrolyte abnormalities, the QTc interval normalized to 422 ms. This clear temporal association, where QT prolongation developed and resolved in parallel with the electrolyte disturbances, provides strong clinical evidence supporting the diagnosis of aLQTS.

Severe hypokalemia is a critical precipitant of aLQTS. In this case, profound hypokalemia (1.7 mmol/L) with hypochloremic metabolic alkalosis (pH 7.71) created a self-perpetuating vicious cycle. Hypokalemia prolongs the cardiac action potential by suppressing the rapid delayed rectifier potassium current (*I_Kr*), while metabolic alkalosis exacerbates hypokalemia via intracellular potassium shifts and potentiates the late sodium current. These combined effects increase repolarization dispersion and lower the threshold for TdP (5, 6).

To interrupt this vicious cycle, management was centered on aggressive yet carefully controlled potassium repletion. Under continuous invasive hemodynamic monitoring, potassium chloride was administered via a central venous catheter at a rate of 0.4 mmol/kg/h. This controlled central administration under continuous monitoring was crucial to mitigate the risks of rapid potassium infusion, such as hyperkalemia or cardiac irritability, while ensuring effective repletion. This approach was designed to safely and rapidly restore extracellular potassium levels and reconstitute myocardial repolarization reserve. Concurrently, 0.9% sodium chloride was administered as the primary intravenous fluid to correct the concomitant hypochloremic metabolic alkalosis.

Magnesium sulfate is a well-established first-line therapy for the prevention and termination of TdP in patients with long QT syndromes (7). In this case, its perioperative use extended beyond correction of coexisting hypomagnesemia. The principal pharmacological rationale lies in its dual action as a myocardial membrane stabilizer and a non-competitive calcium channel antagonist, both of which suppress early afterdepolarizations and elevate the arrhythmic threshold. Accordingly, upon identification of marked QT prolongation, a prophylactic loading dose of magnesium sulfate (40 mg/kg) was administered as a slow intravenous infusion. A single dose was sufficient to achieve normomagnesemia, with serum magnesium rising to 0.85 mmol/L by postoperative day 1. As shown in Figure 3A, the progressive QT shortening closely followed the rise in serum potassium, indicating that sustained recovery was driven by potassium repletion. Magnesium contributed by suppressing arrhythmic risk rather than directly shortening the QT interval.

Effective management of aLQTS requires a comprehensive strategy aimed at minimizing all potential proarrhythmic triggers. In addition to electrolyte correction, a systematic review of all perioperative medications was undertaken to avoid agents known to further prolong the QT interval, including certain fluoroquinolone antibiotics (e.g., moxifloxacin) and 5-hydroxytryptamine type 3 (5-HT3) receptor antagonist antiemetics (e.g., ondansetron) (3). Alternative agents with minimal QT liability were selected whenever possible. We acknowledge that total intravenous anesthesia with propofol might have been preferable, and not using TIVA represents a limitation. During this overnight emergency, our team was more accustomed to inhalational anesthesia in pediatric cases, and the decision to use sevoflurane did not fully account for its potential QT effects. However, several factors mitigated the risk: sevoflurane was maintained at a conservative concentration (0.8–1.0 MAC, age-adjusted), magnesium had been given prophylactically, and aggressive potassium correction was ongoing. As shown in Figure 3A, intraoperative QTc remained stable (fluctuations < ± 25 ms), suggesting sevoflurane’s clinical impact was minimal. This experience highlights an important learning point for future similar cases. Continuous ECG and metabolic monitoring were maintained throughout the perioperative period to mitigate any additional pharmacological or physiological risk of malignant arrhythmia.

Although current guidelines and literature provide detailed recommendations for the treatment of established TdP and for the elective perioperative management of patients with known congenital long QT syndrome (3, 8, 9), this case underscores a more urgent and clinically challenging scenario. Specifically, it underscores the difficulty of preventing life-threatening arrhythmias in a pediatric patient requiring emergency surgery for acute intestinal obstruction in the presence of severe electrolyte derangements and secondary aLQTS. Our experience demonstrates that preemptive intervention, before the onset of TdP, is both feasible and critical when supported by vigilant monitoring and a coordinated, multidisciplinary management strategy.

Systematic exclusion of other potential causes is essential when diagnosing aLQTS. Congenital LQTS was ruled out by the absence of personal or family history, preoperative cardiology consultation revealing no suggestive features, and complete QTc normalization after electrolyte correction. Drug-induced etiology was excluded by thorough medication review. Although concurrent hypocalcemia and hypomagnesemia were present and corrected, the close temporal relationship between potassium normalization and progressive QT shortening (Figure 3A) confirmed hypokalemia as the primary driver. This integrated diagnostic approach strongly supports the diagnosis of aLQTS.

## Conclusion

This case highlights that aLQTS secondary to severe hypokalemia caused by persistent vomiting in toddlers represents an underrecognized but substantial risk factor for perioperative sudden cardiac death. Successful management depended on several key measures: early identification of QTc prolongation as a critical warning signal, prompt prophylactic administration of magnesium sulfate, and simultaneous correction of potassium deficiency and metabolic alkalosis to restore electrophysiological stability. The principal clinical implication is the need to heighten anesthesiologists’ awareness of this condition, promote early detection, and implement structured management protocols for toddlers presenting with vomiting and hypokalemia, thereby preventing potentially fatal arrhythmias.

## Limitations

As a single case report, this study has inherent limitations. Our findings and management approach are specific to this unique scenario and not generalizable. Although the diagnosis of aLQTS is strongly supported by the clinical course, it was not confirmed by genetic testing; therefore, a rare latent form of congenital LQTS cannot be definitively excluded.

## Patient perspective

Initially, our child did not improve with treatment at the first hospital, and we felt extreme anxiety when he was transferred for further care. The diagnosis changed from what was initially believed to be gastroenteritis to a surgical intestinal obstruction, which was frightening for our family. After the anesthesiologist carefully explained the serious risks associated with his severe electrolyte imbalances, including the possibility of dangerous heart rhythm disturbances, we truly understood how critical his condition was. We are deeply grateful to the entire medical team for their expertise and dedication, which ensured our child’s safe recovery and allowed him to return home. We also hope that sharing our experience will raise awareness among other parents about the hidden dangers of superabsorbent polymer toys and the importance of keeping them out of children’s reach.

## Data Availability

The original contributions presented in this study are included in this article/supplementary material, further inquiries can be directed to the corresponding author.
